# Constitutive activation of CTNNB1 results in a loss of spermatogonial stem cell activity in mice

**DOI:** 10.1371/journal.pone.0251911

**Published:** 2021-05-20

**Authors:** Alexandre Boyer, Xiangfan Zhang, Adrien Levasseur, Nour Abou Nader, Guillaume St-Jean, Makoto C. Nagano, Derek Boerboom

**Affiliations:** 1 Centre de Recherche en Reproduction et Fertilité, Faculté de Médecine Vétérinaire, Université de Montréal, Saint-Hyacinthe, QC, Canada; 2 Department of Obstetrics and Gynecology, Division of Reproductive Biology, Faculty of Medicine, McGill University, Montréal, Québec, Canada; Nanjing Medical University, CHINA

## Abstract

Spermatogenesis requires that a careful balance be maintained between the self-renewal of spermatogonial stem cells (SSCs) and their commitment to the developmental pathway through which they will differentiate into spermatozoa. Recently, a series of studies employing various *in vivo* and *in vitro* models have suggested a role of the wingless-related MMTV integration site gene family/beta-catenin (WNT/CTNNB1) pathway in determining the fate of SSCs. However, conflicting data have suggested that CTNNB1 signaling may either promote SSC self-renewal or differentiation. Here, we studied the effects of sustained CTNNB1 signaling in SSCs using the *Ctnnb1*^tm1Mmt/+^; *Ddx4*-Cre^Tr/+^ (Δ*Ctnnb1*) mouse model, in which a stabilized form of CTNNB1 is expressed in all germ cells. Δ*Ctnnb1* mice were found to have reduced testis weights and partial germ cell loss by 4 months of age. Germ cell transplantation assays showed a 49% reduction in total functional SSC numbers in 8 month-old transgenic mice. *In vitro*, *Thy*1-positive undifferentiated spermatogonia from Δ*Ctnnb1* mice formed 57% fewer clusters, which was associated with decreased cell proliferation. A reduction in mRNA levels of genes associated with SSC maintenance (*Bcl6b*, *Gfra1*, *Plzf*) and increased levels for markers associated with progenitor and differentiating spermatogonia (*Kit*, *Rarg*, *Sohlh1*) were detected in these cluster cells. Furthermore, RNAseq performed on these clusters revealed a network of more than 900 genes regulated by CTNNB1, indicating that CTNNB1 is an important regulator of spermatogonial fate. Together, our data support the notion that CTNNB1 signaling promotes the transition of SSCs to undifferentiated progenitor spermatogonia at the expense of their self-renewal.

## Introduction

Spermatogenesis is sustained by the spermatogonial stem cells (SSCs); a cell population that can be directed to either self-renew to replenish the SSC pool, or to differentiate to eventually become spermatozoa. This fate decision is tightly regulated during steady-state spermatogenesis. Although this process is poorly understood, it was shown that glial-cell-line-derived neutrophic factor (GNDF) [1, 2] is a prominent factor that promotes the maintenance of the SSC pool, whereas retinoic acid (RA) induces spermatogonial differentiation [3–5]. In agreement with this, it was shown that stem cell function resides nearly exclusively within a subpopulation of type A undifferentiated spermatogonia that strongly expresses GDNF receptor alpha 1 (GFRA1) [6]. These GFRA1-positive cells differentiate into neurogenin 3 (NGN3)/ retinoic acid receptor gamma (RARG)-expressing cells, which in turn transition into differentiating spermatogonia that express kit oncogene (KIT) [7]. Aside from GDNF and RA, other factors such as fibroblast growth factor 2 (FGF2) [8, 9], colony stimulating factor 1 (CSF1) [10], kit ligand (KITL) [11, 12] and members of the wingless-related MMTV integration site (WNT) gene family [13, 14] have all been shown to regulate these processes.

WNT ligands can signal via canonical and non-canonical pathways. The canonical (or WNT/beta-catenin (CTNNB1)) pathway acts to inhibit the degradation and promote the nuclear translocation of CTNNB1, which subsequently associates with transcription factors to modulate the transcriptional activity of target genes [15]. Non-canonical signaling mechanisms include the WNT/calcium and planar cell polarity pathways, which either act independently of CTNNB1 or can serve to antagonize canonical signaling in certain contexts [16, 17]. In the last decade, potential roles for WNT signaling in the regulation of SSCs have been reported. It was first suggested that non-canonical and canonical pathways have opposite effects on SSC maintenance, as non-canonical WNT5A promoted SSC survival *in vitro*, whereas activation of CTNNB1 with lithium chloride (LiCl) lead to a decrease in SSC numbers [13]. A subsequent study using a similar culture model showed that activation of CTNNB1 with WNT3A stimulated the proliferation of progenitor spermatogonia already committed to differentiation, but that cells expressing CTNNB1 had only minimal SSC activity [14]. An *in vivo* study showed that inactivation of *Ctnnb1* in undifferentiated spermatogonia lead to a reduction in their total number without affecting the pool of GFRA1-positive cells, suggesting that CTNNB1 is important for the differentiation or proliferation of undifferentiated spermatogonia, but not for the maintenance of the SSC pool [18]. Other studies examined the effects of constitutive activation of canonical signaling *in vivo* through conditional expression of a dominant-stable CTNNB1 mutant in gonocytes. Two groups generated a *Ctnnb1*^tm1Mmt/+^; Ddx4-Cre^Tr/+^ mouse model (hereafter Δ*Ctnnb1*) in which recombination occurs in gonocytes between e15.5 and birth [19, 20]. One group observed robust proliferation of undifferentiated spermatogonia followed by apoptosis of the spermatocytes in this model, and GFRA1 expression was not affected [20]. This lead to the loss of germ cells in 40% of the seminiferous tubules in one month-old animals, increasing to 45% at 3 months of age [20]. The second group rather observed that spermatogenesis was not affected before 13 weeks of age, but found spermatogenesis to be defective in 40% of the seminiferous tubules at 75 weeks of age, with reduced germ cell proliferation and loss of pre-leptotene germ cells [19]. Lastly, a third group used a distinct model (*Ctnnb1*^tm1Mmt/tm1Mmt^;*Nanos3*^cre/+^) in which recombination in gonocytes was complete by e14.5. In these mice, spermatogenesis was defective in 20% of the seminiferous tubules of animals aged between two and four months, which was associated with a reduction in the number of GFRA1-positive cells [21]. The differences observed among the aforementioned studies therefore indicate that the role of CTNNB1 signaling in male germ cell biology remains incompletely understood, particularly with regards to its effects in stem and progenitor cell populations.

In the present study, we reexamined the Δ*Ctnnb1* model, paying particular attention to the global effects of CTNNB1 on the undifferentiated spermatogonia and SSC populations.

## Material and methods

### Ethics

All animal procedures were approved by the Comité d’Éthique de l’Utilisation des Animaux of the Université de Montréal (protocol number 1320) and conformed to the Canadian Council on Animal Care.

### Transgenic mouse strains

FVB-Tg(Ddx4-cre)1Dcas/J (*Ddx4*-Cre^Tr/+^) and *B6*.*129S7-Gt(ROSA)26Sor/J* (*Rosa26*^Sor^) mice were obtained from the Jackson Laboratory (Bar Harbor, ME). *Ctnnb1*^tm1Mmt/+^ mice, harboring a *Ctnnb1* allele with a loxP-flanked third exon [22], were a kind gift from Dr Makoto Taketo (Kyoto University, Japan). *Ctnnb1*^tm1Mt/+^ were crossed with *Ddx4*-Cre^Tr/+^ to obtain the *Ctnnb1*^tm1Mmt/+^;*Ddx4*-Cre^Tr/+^ (ΔCtnnb1) genotype. *Ddx4*-Cre^Tr/+^ mice were not used for breeding after 8 weeks of age due to potential Cre accumulation in the germ cells, zygotic recombination or leaking [23]. *Ctnnb1*^tm1Mmt/+^ mice were also crossed with the *Rosa26*^Sor^ strain to obtain *Rosa26*^Sor/Sor^;*Ctnnb1*^tm1Mmt/tm1Mmt^ mice, which were subsequently mated to *Ddx4*-Cre^Tr/+^ to obtain the *Rosa26*^Sor/+^;*Ctnnb1*^tm1Mmt/+^;*Ddx4*-Cre^Tr/+^ genotype (hereafter *Rosa*-Δ*Ctnnb1*). Genotype analyses were done on tail biopsies by PCR as previously described for *Ctnnb1* [22] or *Cre* recombinase and *Rosa26* (Jackson laboratory, Cre generic protocols and Gt(ROSA)26Sor standard PCR assay). PCR products were analyzed by electrophoresis using a 2% agarose gel containing ethidium bromide and photographed under UV illumination.

### Histopathology and immunofluorescence

Testes for light microscopy histopathologic analysis were weighed, fixed in Bouin’s solution for 24 h, rinsed, and dehydrated in alcohol. Tissues were embedded in paraffin, sectioned, and stained with hematoxylin and eosin. For immunofluorescence experiments, fresh testes were embedded in OCT compound (Sakura Finetek, Torrance, CA) and stored at −80°C before the preparation of 3μm sections, which were fixed for 30 min in PBS-buffered 4% paraformaldehyde at room temperature, followed by overnight in PBS-buffered 1% paraformaldehyde at 4°C. Sections were labeled with a primary antibody against CTNNB1 (8480, Cell Signaling, Danvers, MA) in PBS/0.3% Triton X-100 overnight at 4°C and subsequently probed with secondary Alexa Fluor 594 goat anti-rabbit (Molecular Probes, Eugene, OR) for 1 h at room temperature. Slides were mounted using VectaShield with 4′,6-diamidino-2-phenylindole (DAPI) (Vector Labs). Photomicrographs were taken using a Carl Zeiss Axio Imager M1 microscope (Carl Zeiss Canada Ltd, Toronto, Canada) using Zen 2012 blue edition software (Carl Zeiss, Oberkochen, Germany).

### Germ cell transplantations

Donor cell suspensions were prepared from testes of 8 month-old *Rosa*-Δ*Ctnnb1* and *Rosa26*^Sor/+^;*Ctnnb1*^tm1Mmt/+^ (hereafter *Rosa*-control) mice using a standard two-step enzymatic digestion protocol [24], except that 1 mg/ml of collagenase I, 1 mg/ml collagenase IV, 1 mg/ml hyaluronidase and 1 mg/ml DNase I (Sigma, St. Louis, MO) were used in the first step [25]. Transplant recipients were wild-type 129/SvEv×B6 F_1_ hybrid mice. To deplete endogenous spermatogenesis, recipient mice were treated with busulfan (50 mg/kg, i.p.) at 6 weeks of age [24]. Six weeks later, donor cells were resuspended at 1.0–1.2×10^6^ cells/ml, and injected into recipient seminiferous tubules through the efferent duct as previously described [24]. For SSC quantification, recipient testes were harvested 8 weeks after transplantation, and stained with X-gal to visually count the colonies of donor-derived spermatogenesis as previously described [26].

### Thy1-positive spermatogonia culture and cluster analysis

Cluster cultures, in which SSCs are maintained long-term *in vitro* with an exponential propagation [25, 26], were generated from immunomagnetically-selected Thy1-positive testis cells from 6 day-old *Rosa*-Δ*Ctnnb1* and *Rosa*-control mice, and cultured on STO feeder cells that had been mitotically inactivated with mitomycin C as previously described [27, 28]. The SSC medium was composed of Minimum Essential Medium alpha (Thermo Fisher scientific), with 0.2% BSA (Sigma-Aldrich, Oakville, Ontario, Canada) and supplements as previously described [27, 28]. The growth factors used were recombinant human GDNF (R&D Systems, Minneapolis, MN), recombinant rat GFRA1 (R&D Systems) and FGF2 (Thermo Fisher scientific) at 40 ng/ml, 300 ng/ml, and 1 ng/ml respectively [27, 28]. Under these conditions, spermatogonia develop into cell aggregates, termed “clusters”, by day 6 after seeding. Media and growth factors were replenished on day 3 and clusters were subcultured onto freshly prepared STO feeder cells in a 6- to 7-day interval [27, 28]. Once established (5 passages), clusters were maintained under reduced growth factor (rGF) conditions, with 20 ng/ml GDNF, 75 ng/ml GFRA1, and 1 ng/ml FGF2. All cultures were maintained at 37°C in a humidified incubator with 5% CO_2_. RNA extraction was done using clusters obtained after 6–8 passages; trypsinized single cells (7500 cells/well) were seeded in three wells (24-well plates) and allowed to form clusters. After seven days of culture, clusters were pooled from the three wells for RNA extraction. Cluster counting was done using clusters obtained after 11–15 passages; trypsinized single cells (7500 cells/well) were seeded in one well and clusters were counted following fixation and X-gal staining as previously described [13].

### Analyses of cell death, cell cycle and Kit+ cells

To examine cell death and cell cycle profiles of cluster cells, clusters were removed from STO feeder cells by gentle pipetting [29] and trypsinized into single cells. Single cells were then seeded onto a new STO feeder layer at 2 x 10^5^ cells/well in a 24-well plate and cultured in rGF medium for 6 days, with a medium change on day 3. On day 6, clusters were harvested by gently pipetting, trypsinized into single cells and subjected to cell death analysis. Approximately ~5 x 10^5^ harvested cells were resuspended in PBS and incubated with 1μg/ml propidium iodide (PI) (Sigma Aldrich) for 1 minute at room temperature in the dark. PI fluorescence was determined using an Accuri™ C6 flow cytometer (BD Biosciences, Franklin lakes, NJ). Data were collected from three experiments with 50,000 events collected per sample. For the cell cycle experiment, harvested cells (~10^6^) were fixed with 70% ice-cold ethanol overnight, followed by an incubation with 40 μg/ml PI and 100 μg/ml RNase A (Thermo Scientific) at 37°C for 30 minutes. Stained nuclei were analyzed with a FACSCanto™ II (BD Biosciences) for 10000 events per sample. Collected data from triplicate experiments were analyzed by ModFit LT™ software (Verity Software House, Topsham, ME).

To determine the percentage of differentiated spermatogonia contained within the clusters, day 6 clusters were removed from STO feeder cells by gentle pipetting [29] and dissociated into single cells using an enzyme-free cell dissociation buffer (Thermo Scientific). ~0,2 x 10^5^ cells for the *Rosa*-Δ*Ctnnb1* and 2 x 10^5^ cells for the *Rosa*-control were then incubated with a brilliant violet 421 c-kit antibody (105827, BioLegend, San Diego, CA) for 30 minutes. Numbers of stained cells was determined using an LSRFortessa X-20 flow cytometer (BD Biosciences). Data were collected from three experiments.

### Reverse transcription PCR (RT-PCR) and reverse transcription-quantitative PCR (RT-qPCR)

A two-step RT-PCR to detect expression of *Ctnnb1* from the recombined *Ctnnb1*^tm1Mmt^ allele was performed on 1μg RNA samples purified from testes of Δ*Ctnnb1* and *Ctnnb1*^tm1Mmt/+^ (hereafter control) mice using the RNeasy mini kit (Qiagen) according to the manufacturer’s protocol. RT was performed using the Superscript one-step RT-PCR kit (Thermo Fisher scientific) as previously described [30]. For RT-qPCR experiments, total RNA from cultured spermatogonial stem cells (SSCs) was extracted using the RNeasy mini kit (Qiagen). Total RNA (100 ng) was reverse transcribed using the SuperScriptVilo cDNA synthesis kit (Thermo Fisher scientific). Real-time PCR reactions were run on a CFX96 Touch instrument (Bio-Rad, Hercules, CA), using Supergreen Advanced qPCR MasterMix (Wisent, St-Bruno, Canada). Each PCR reaction consisted of 7.5 μl of Power SYBR Green PCR Master Mix, 2.3 μl of water, 4 μl of cDNA sample, and 0.6 μl (400 nM) of gene-specific primers. PCR reactions run without complementary cDNA (water blank) served as negative controls. A common thermal cycling program (3 min at 95°C, 40 cycles of 15 s at 95°C, 30 s at 60°C and 30 s at 72°C) was used to amplify each transcript. To quantify relative gene expression, the Ct of genes of interest was compared with that of ribosomal protein L19 (*Rpl19*), according to the ratio R = [E^Ct *Rpl19*^/E^Ct target^], where E is the amplification efficiency for each primer pair. *Rpl19* Ct values did not change significantly between tissues or cells, and *Rpl19* was therefore deemed suitable as an internal reference gene. The specific primer sequences used for RT-qPCR are listed in Table 1.

**Table 1:** Quantitative RT-qPCR primer sequences.

Gene	Forward	Reverse
***Axin2***	GAGGTGGTACCTTGCCAAAA	TTCCTGTCCCTCTGCTGACT
***Bcl6b***	TTCTTATCGCTTGCAGTGGCT	GAGAGAGTACATCCACCCCGA
***En1***	CCGGTGGTCAAGACTGACTC	CTGGTGCGTGGACCAGAG
***Eomes***	ACCAAAACACGGATATCACCC	TAGTTGTCCCGGAAGCCTTTG
***Gfra1***	CACTCCTGGATTTGCTGATGT	AGTGTGCGGTACTTGGTGC
***Hoxa9***	TCTCCGAAAACAATGCCGAGA	GCAGCCGGGTTATTGGGA
***Kit***	TGGAGTTTCCCAGAAACA	AAATGGGCACTTGGTTTGA
***Lef1***	GACGAGCACTTTTCTCCGGG	TGGGGTGATCTGTCCAACGC
***Msx1***	CCGAAAGCCCCGAGAAACTA	GACTCAGCCGTCTGGC
***Onecut2***	AGAGGGTTCTATGCCGGTCT	CTTTGCGTTTGCATGCTGCC
***Pax2***	AACCCGACTATGTTCGCCTG	TTGGTCCGGATGATCCTGTTG
***Plzf***	GCAGCTATATTTGCAGTGA	TCTTGAGTGTGCTCTCATCC
***Rarg***	CTCGGGTCTATAAGCCATGC	CCCCATAGTGGTAGCCAGAA
***Rpl19***	CTGAAGGTCAAAGGGAATGTG	GGACAGAGTCTTGATGATCTC
***Sohlh1***	GACCCTGAATCTTCCGGCAT	TGTGGTGTACCTGGCATCA
***Vsx1***	AAGAATGACCCGAAGATGTCCC	CAGTGAAAACCGTCCTGTGCC
***Wt1***	AGCTGTCCCACTTACAGATGCAT	GGATGCTGGACTGTCTCCGTGT

### RNAseq analyses

Total RNA was extracted from cluster cells using the RNeasy mini kit (Qiagen) according to the manufacturer’s protocol and submitted to the genomics core facility of the Institute for Research in Immunology and Cancer (IRIC, Montreal, Quebec, Canada). RNA quality and concentration were determined with an Agilent 2100 Bioanalyzer using the RNA 6000 Pico kit (Agilent Technologies). Six RNA-seq libraries (n = 3 cluster cells from *Rosa*-Δ*Ctnnb1* and n = 3 from *Rosa*-control) were generated using the KAPA mRNA Hyperprep (poly-A capture) libraries (Roche, Mississauga, Ontario, Canada). Single-read (1X 75 base pairs, maximum 1X 85bp) sequencing was performed on a Nextseq500- 0.5 Flowcell High Output (40 M reads per sample) (Illumina, San Diego, CA). Sequences were trimmed of sequencing adapters and low quality 3’ bases using Trimmomatic version 0.35 [31] and aligned to the reference mouse genome version GRCm38 (gene annotation from Gencode version M23, based on Ensembl 98) using STAR version 2.7.1a [32]. Gene expression was obtained both as readcount directly from STAR as well as computed using RSEM [33] in order to obtain normalized gene and transcript level expression in TMP values for these non stranded RNA libraries. DeSeq2 version 1.22.2 [34] was then used to normalize gene readcount. Results were further analyzed using the Metascape gene annotation and analysis resource to evaluate the biological processes regulated by the up or downregulated genes (2-fold or more) [35] or processed through Cytoscape [36] using GeneMANIA [37] to evaluate their possible interactions with CTNNB1 (top 50 upregulated and downregulated genes). *Ctnnb1* was added to the list of genes to better evaluate their interaction with *Ctnnb1*. Visual representation of the network connectivity with *Ctnnb1* was performed using the neighbor’s option on the *Ctnnb1* node.

### Statistical analyses

All statistical analyses were performed with Prism software version 6.0d (GraphPad Software Inc.). Student’s t-test was used to determine effects of genotype in the data shown in all figures. All the data sets were subjected to the F test to determine the equality of variances prior to statistical testing. Means were considered significantly different when P values were <0.05. All data are presented as means ± standard error of the mean.

## Results

### Constitutive activation of CTNNB1 in spermatogonia induces progressive germ cell loss in the Δ*Ctnnb1* mouse model

To evaluate the role of CTNNB1 in early germ cell development, a strategy was devised to constitutively activate CTNNB1 in prospermatogonia by crossing *Ctnnb1*^tm1Mmt/tm1Mt^ mice with the *Ddx4*-cre strain. The *Ctnnb1*^*tm1Mmt*^ strain carries a *Ctnnb1* allele whose third exon is flanked by *loxP* sites. Upon recombination, the *Ctnnb1*^*tm1Mmt*^ allele produces a mutant CTNNB1 protein that, although functional, lacks a series of phosphorylation sites that are required for its degradation, resulting in its accumulation and translocation to the nucleus [22]. The *Ddx4*-cre strain is known to drive Cre-mediated recombination in germ cells starting at embryonic day e15.5, with Cre-mediated recombination occurring in > 95% of germ cells by the time of birth [23].

**Fig 1:**
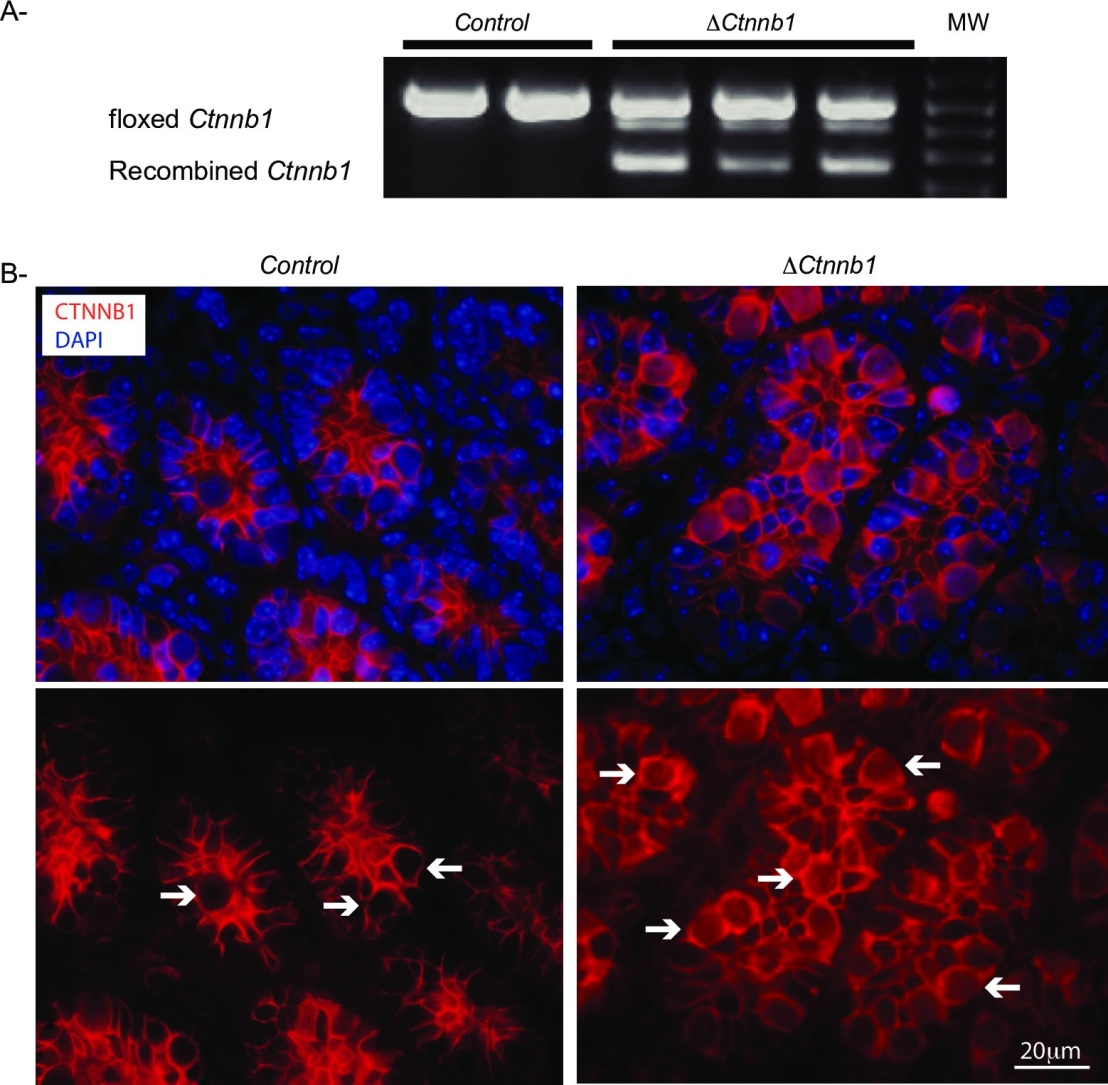
CTNNB1 stabilization in Δ*Ctnnb1* mice. **A)** RT-PCR analyses of *Ctnnb1* in testes from 5 day-old Δ*Ctnnb1* and control mice. As indicated, a shorter RT-PCR fragment is produced from transcripts derived from the recombined *Ctnnb1*^tm1Mmt^ allele. **B)** Immunofluorescence analysis of CTNNB1 expression in testes from 5 day-old Δ*Ctnnb1* and control mice. Scale bar (lower right) is valid for all images. Arrow = germ cells.

To evaluate the efficiency of the Cre-mediated recombination, we first performed RT-PCR analyses on RNA extracted from testes of 5 day-old Δ*Ctnnb1* and control mice. An RT-PCR product derived from the Cre-recombined RNA was readily detected in the testes of mutant animals, indicating that recombination had already occurred at 5 days of age (Fig 1A). To confirm the constitutive activation of CTNNB1 in germ cells, immunofluorescence analyses were done on testes from 5 day-old mice. As expected, CTNNB1 was detected almost exclusively at the membrane of both Sertoli cells and germ cells in controls, but was also detected in the cytoplasm and the nucleus of germ cells in Δ*Ctnnb1* animals (Fig 1B). Taken together, these results indicate that stabilization of CTNNB1 was obtained in germ cells of Δ*Ctnnb1* mice.

**Fig 2:**
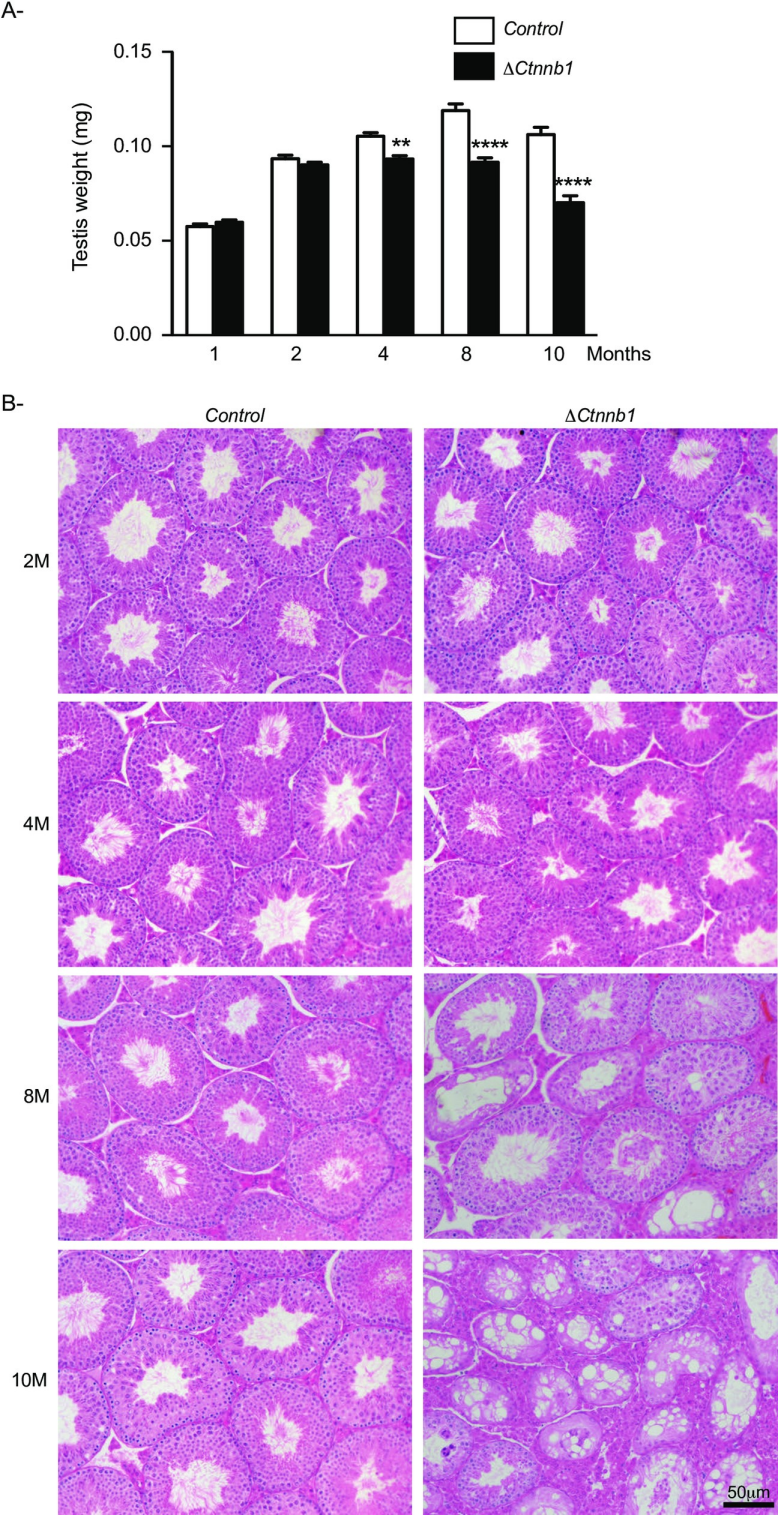
Loss of spermatogenesis in the seminiferous tubules of Δ*Ctnnb1* mice. **A)** Time course analysis of testicular weight comparing Δ*Ctnnb1* to control mice at the indicated ages. Sample numbers analyzed varied by age and genotype. Values for Δ*Ctnnb1* are 1M: n = 4; 2M: n = 6; 4M: n = 14; 8M: n = 8; 10M: n = 14; values for control are 1M: n = 4; 2M: n = 8; 4M: n = 18; 8M: n = 8; 10M: n = 12. Data are expressed as means (columns) ± SEM (error bars). Asterisks indicate significant differences from controls (** *P* **<** 0.01; **** *P* < 0.0001)**. B)** Photomicrographs comparing testicular histology of Δ*Ctnnb1* to control mice at the indicated ages. Scale bar (lower right) is valid for all images; Hematoxylin and Eosin staining.

To evaluate the effect of the constitutive activation of CTNNB1 in male germ cells on subsequent spermatogenesis, testes from Δ*Ctnnb1* mice were evaluated at 1, 2, 4, 8 and 10 months of age. Time course analysis of the Δ*Ctnnb1* mice showed a progressive atrophy of the testes starting at 4 months of age (Fig 2A). By 10 months of age, testes of Δ*Ctnnb1* mice decreased to ≈ 70% of normal weight (Fig 2A). Histopathologic assessment of the testes from 1-, 2- and 4 month-old Δ*Ctnnb1* mice showed them to be indistinguishable from those of age-matched controls (Fig 2B), despite the small reduction in testis weights observed in 4 month-old mutant mice. However, at 8 months of age, loss of germ cell layers was evident in a few tubules in the testes of Δ*Ctnnb1* mice (Fig 2B). By 10 months of age, most of the tubules showed signs of loss of spermatogenesis (Fig 2B). This gradual loss suggested the inability to replenish differentiating spermatogenic cells and the depletion of the SSC population over time. These results are similar to the ones observed by Kumar et al. [19] but differ from the results published by Chassot et al. [20], in which loss of germ cells was already apparent at 15 days postpartum, and affected more than 40% of the tubules by 1 month of age [20]. The histologic changes that we observed were also similar to those reported to occur in the *Ctnnb1*^tm1Mmt/tm1Mmt^;*Nanos3*^cre/+^ model [21].

### Constitutive activation of CTNNB1 promotes spermatogonial stem cell differentiation

**Fig 3:**
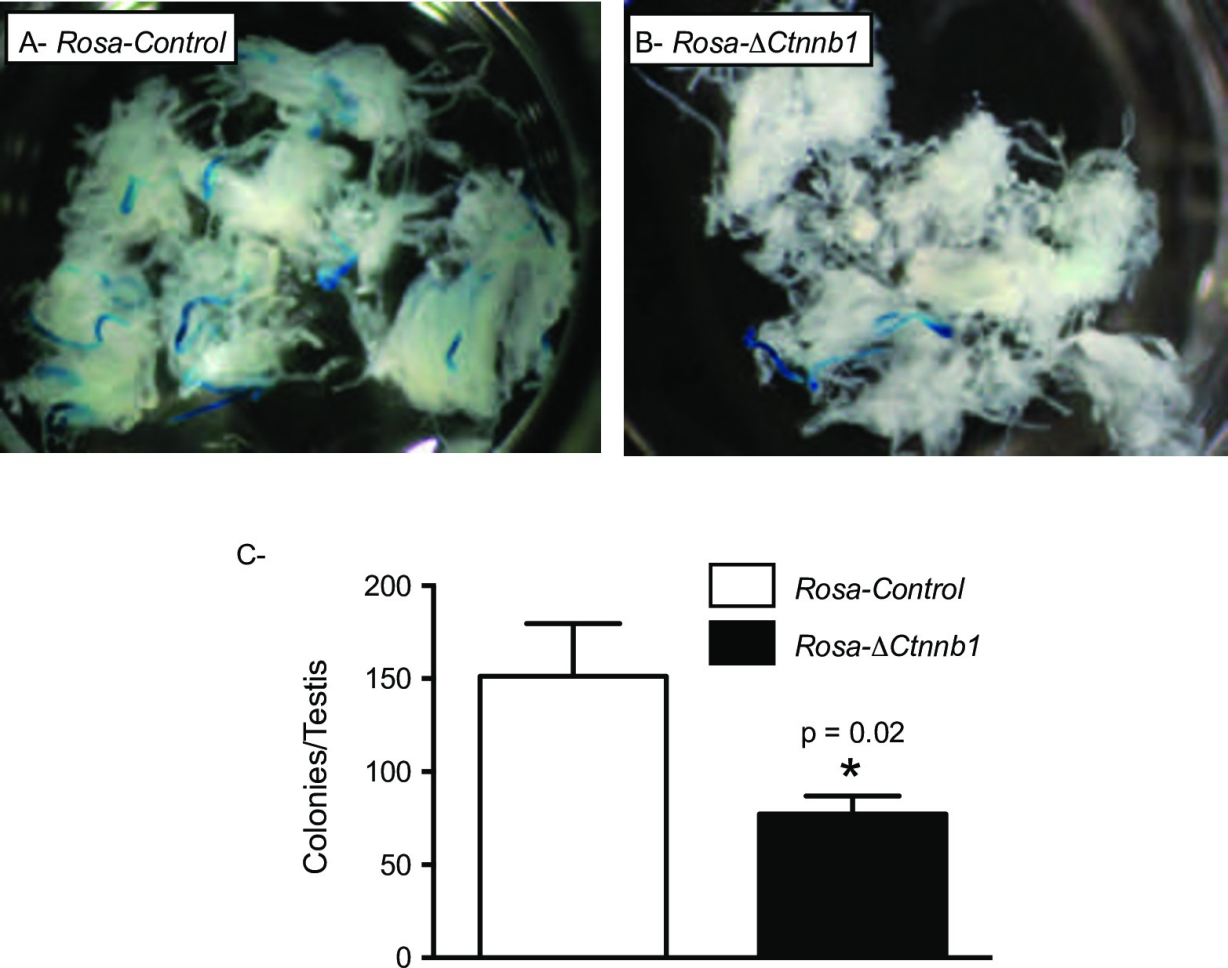
Δ*Ctnnb1* mice lose spermatogonial stem cell activity. **A, B)** Photographs of decapsulated, LacZ-stained recipient testes 8 weeks after transplantation of donor cells from 8-month-old (A) *Rosa-*control and (B) *Rosa*-Δ*Ctnnb1* mice. Blue tubule segments represent colonies of donor-derived spermatogenesis. **C)** Total numbers of functional spermatogonial stem cells present in a donor testis, calculated by multiplying colony numbers (colonies per million transplanted cells) by the total number of germ cells harvested from a donor testis (n = 4 *Rosa-*control and n = 5 *Rosa*-Δ*Ctnnb1* donor animals whose cells were transplanted in 13 recipient testes/genotype). Data are expressed as means (columns) ± SEM (error bars). * Significant difference from control (*P* = 0.02).

Because most available markers for SSCs (such as GFRA1) are also detected in A_paired_ and A_aligned_ undifferentiated spermatogonia [38, 39], the only unequivocal test of SSC activity currently available is the germ cell transplantation assay. In this assay, germ cells from a donor animal are transplanted into a germ cell-depleted recipient, leading to the colonization of the SSC niche by the donor SSCs and the regeneration of spermatogenesis. We therefore used this approach to determine if germ cell loss in Δ*Ctnnb1* mice could be associated with a loss in SSC activity. To perform this test, the *Rosa26*^Sor^ transgene (which expresses the *Escherichia coli lacZ* transgene in all cell types including germ cells) was bred into the Δ*Ctnnb1* strain to permit the visualization of spermatogenesis derived from the donor cells after transplantation *in vivo*. Germ cells from 8 month-old *Rosa*-Δ*Ctnnb1* and *Rosa-*control donor mice were transplanted into germ cell-depleted wild-type mice and the recipient testes were analyzed two months later. A 49% reduction in total functional SSC numbers per testis (Fig 3A–3C) was observed, suggesting that the germ cell loss observed in the Δ*Ctnnb1* model is associated with a loss of SSC activity.

**Fig 4:**
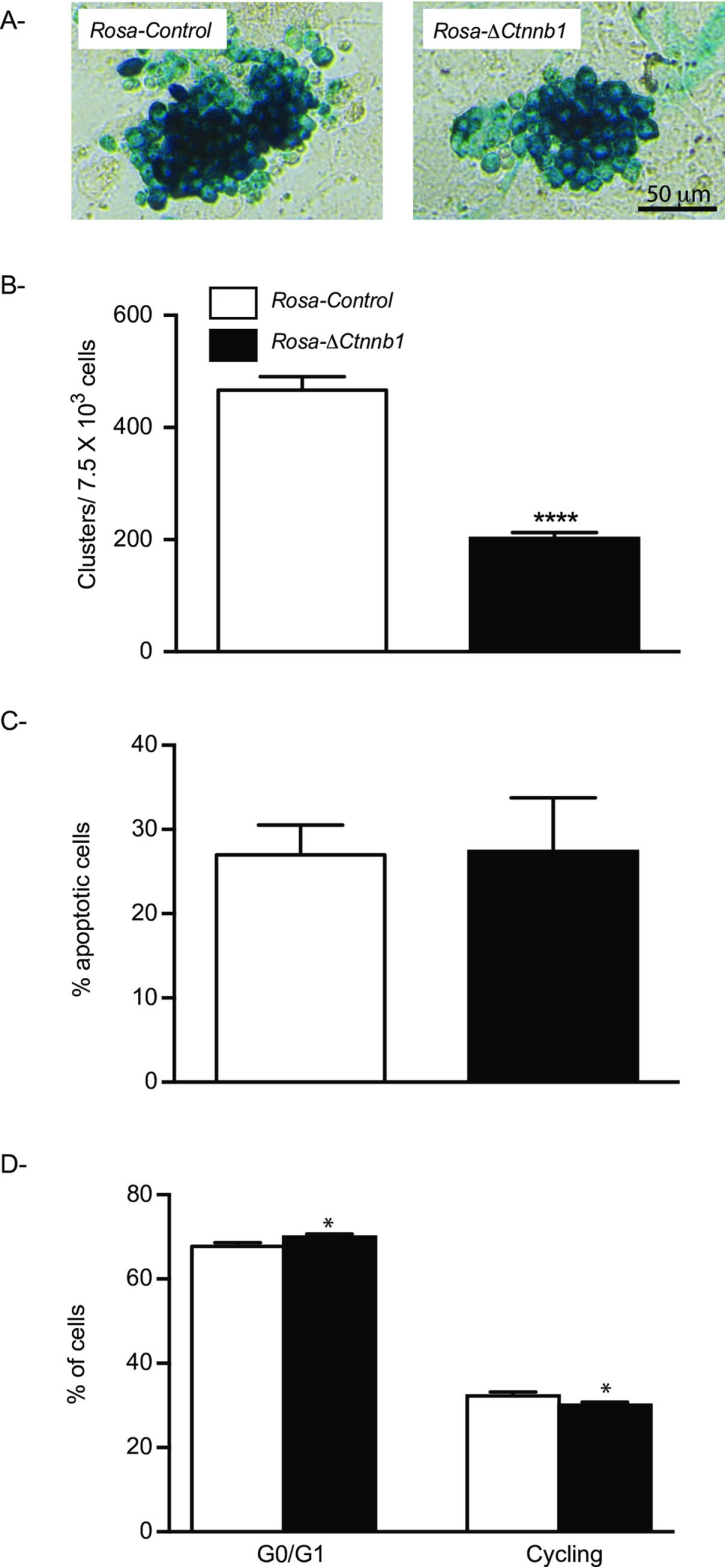
CTNNB1 stabilization inhibits germ cell cluster-forming activity *in vitro*. **A)** Representative photographs of germ cell clusters derived from *Rosa*-Δ*Ctnnb1* and *Rosa-*control mice after 7 days of culture. **B)** Quantification of cluster formation from Thy1-positive cells derived from *Rosa*-Δ*Ctnnb1* and *Rosa-*control mice (n = 6). The results indicate the number of clusters per 7.5 x 10^3 cells initially placed in culture. **C)** Cell death evaluation by propidium iodide incorporation in cultured spermatogonia derived from *Rosa*-Δ*Ctnnb1* and *Rosa-*control mice (n = 3). **D)** Cell cycle profiles of cultured spermatogonia derived from *Rosa*-Δ*Ctnnb1* and *Rosa-*control mice (n = 3). All data are expressed as mean (columns) ± SEM (error bars). Asterisks indicate significant differences from controls (*P < 0.05; **** *P* < 0.0001).

To better determine the cause of the loss of SSC activity in Δ*Ctnnb1* mice, testis cells were enriched for SSCs and cultured on feeder cells in presence of GDNF, GFRA1 and FGF2, resulting in the formation of undifferentiated spermatogonial aggregates (clusters). Although SSCs represent only a small fraction of cluster cells, the number of clusters semi-quantitatively and linearly correlates with the number of SSCs [27, 40]. Following establishment of stable THY1+ undifferentiated spermatogonia cultures from *Rosa*-Δ*Ctnnb1* and *Rosa-*control mice, cluster-forming ability of the undifferentiated spermatogonia was assessed as an *in vitro* indicator of SSC activity. Stabilization of CTNNB1 reduced cluster formation by 57% (Fig 4B), mirroring the reduction in SSC activity observed in the *in vivo* transplantation assay. Apoptosis and cell cycle profiles of the cells in the clusters were then examined by propidium iodide (PI) incorporation. Percentages of PI-positive cells were similar between the clusters derived from the mutant and the control animals (Fig 4C), suggesting that cell death did not lead to the loss of SSC activity. Examination of the cell cycle profile by FACS analysis showed a small but statistically significant increase in the proportion of cells in the G0/G1 phase of cell cycle and a concomitant decrease in cells in the G2/S/M phase in the clusters derived from the mutant animals (Fig 4D).

**Fig 5:**
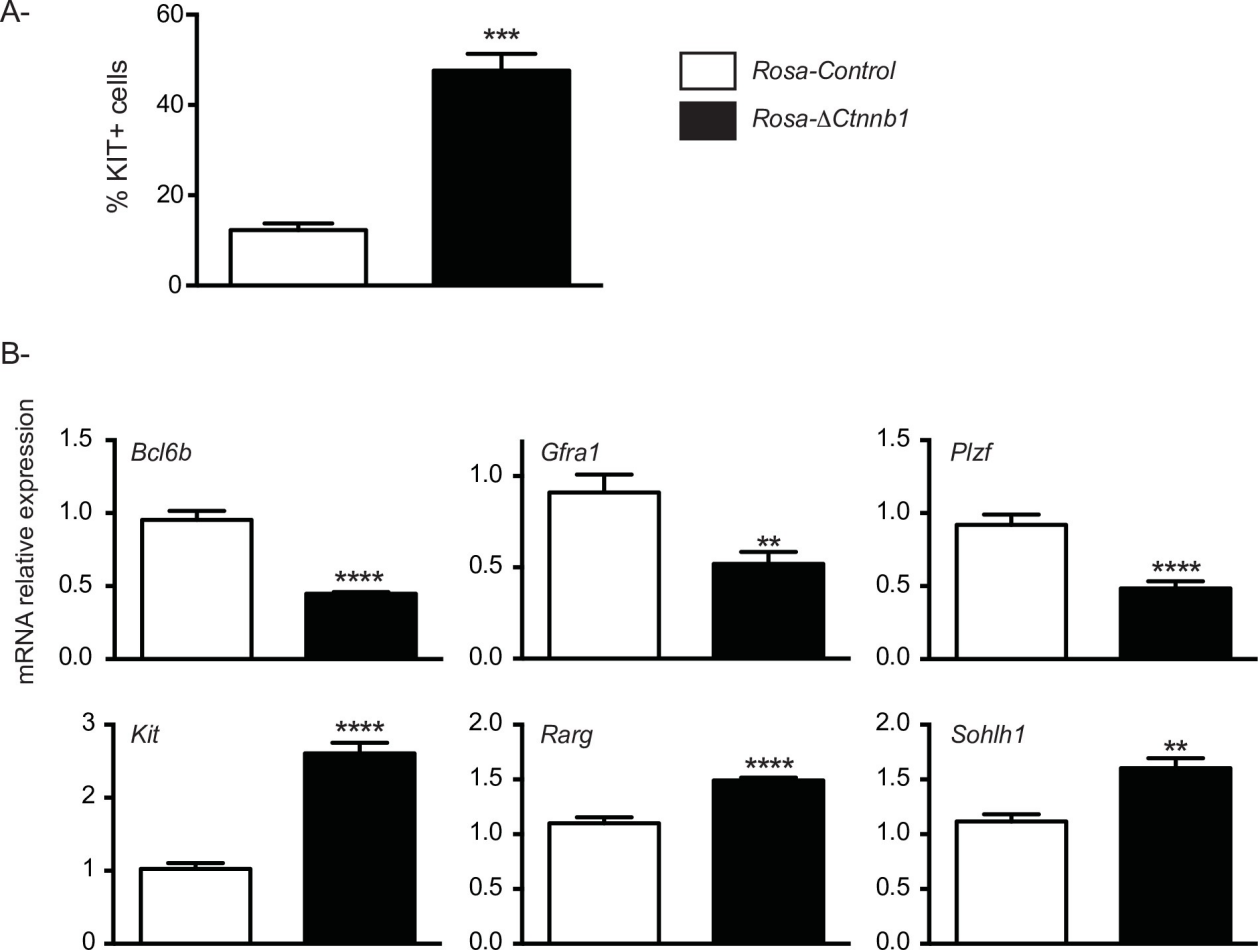
CTNNB1 stabilization skews cluster cell fate towards differentiation. **A)** Evaluation of the number of KIT+ cells in Thy1-positive cell clusters derived from *Rosa*-Δ*Ctnnb1* mice and *Rosa-*control mice (n = 3). **B)** RT-qPCR analysis of the expression of genes involved in SSC maintenance or their transition to undifferentiated progenitor spermatogonia in clusters derived from *Rosa*-Δ*Ctnnb1* and *Rosa-*control mice (n = 10 for *Rosa-*control and n = 11 *Rosa*-Δ*Ctnnb1* for *Gfra1*, *Plzf* and *Kit* and n = 6 /genotype for *Bcl6b*, *Rarg* and *Sohlh*). RT-qPCR data were normalized to the housekeeping gene *Rpl19*. All data are expressed as mean (columns) ± SEM (error bars). Asterisks indicate significant differences from controls (**P < 0.01; *** *P* < 0.001; **** *P* < 0.0001).

To determine if CTNNB1 stabilization skewed SSC cell fate toward their commitment to progenitor spermatogonia in cluster cells established from *Rosa*-Δ*Ctnnb1* mice, the proportion of KIT+ cells in the clusters was evaluated by flow cytometry. In cultures established from *Rosa-*control mice, 12% of the cells forming the clusters were KIT+, while the proportion of KIT+ cells increased to 48% in the clusters established from *Rosa*-Δ*Ctnnb1* mice (Fig 5A). Expression of genes important for SSC maintenance or their transition to undifferentiated progenitor spermatogonia were then evaluated in clusters derived from mice of both genotypes. After 7 days of culture, levels of markers associated with SSC self-renewal (B cell CLL/lymphoma 6, member b, (*Bcl6b*), *Gfra1* and promyelocytic leukemia zinc finger, *Plzf*) were decreased (Fig 5B), whereas expression of key genes associated with the transition to undifferentiated progenitor spermatogonia (*Kit*, retinoid acid receptor gamma, *Rarg* and spermatogenesis and oogenesis specific basic helix-loop-helix 1, *Sohlh1*) increased in the cluster cells from *Rosa*-Δ*Ctnnb1* mice (Fig 5B), again suggesting that CTNNB1 promotes the commitment of SSCs towards their transition to progenitor spermatogonia. Taken together, the reduced cluster formation, proportion of KIT+ positive cells and gene expression patterns suggest that stabilization of CTNNB1 skews the fate of SSCs toward their transition to undifferentiated progenitor spermatogonia.

### CTNNB1 regulates a large network of genes in spermatogonia

**Fig 6:**
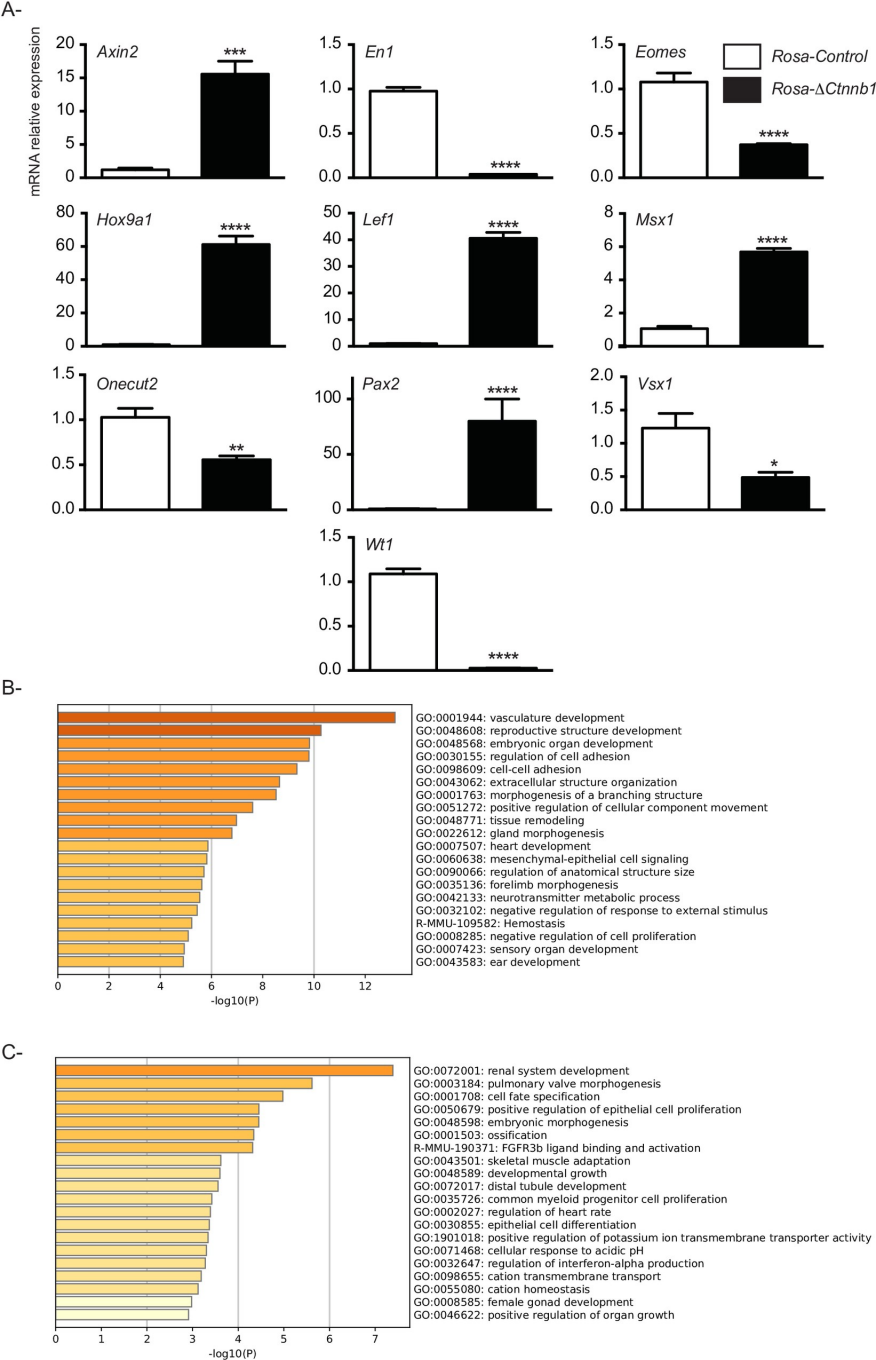
CTNNB1 stabilization mainly affects developmental gene expression. **A)** Validation of the RNAseq data by RT-qPCR analysis (n = 6 for *Rosa-*control and n = 6 *Rosa*-Δ*Ctnnb1*). RT-qPCR data were normalized to the housekeeping gene *Rpl19*. All data are expressed as mean (columns) ± SEM (error bars). Asterisks indicate significant differences from controls (*P < 0.05; **P < 0.01; *** *P* < 0.001; **** *P* < 0.0001). **B, C)** Biological processes associated with upregulated (**B**) or downregulated (**C**) genes in Thy1-positive cell clusters derived from *Rosa*-Δ*Ctnnb1* mice using Metascape.

To determine how the stabilization of CTNNB1 affected the transcriptome of spermatogonia, cluster cells derived from *Rosa*-Δ*Ctnnb1* and *Rosa-*control mice were analyzed by RNAseq. These analyses identified 911 coding genes (minimum of 10 reads in either mutant or control samples) that were differentially expressed between the two groups by 2-fold or more (S1 Table (50 most upregulated genes), 2 (50 most downregulated genes), 3 (raw data)). To validate the data set, mRNA levels of selected genes identified as highly changed (*Rosa*-Δ*Ctnnb1* vs *Rosa-*control) by RNAseq were verified by RT-qPCR (Fig 6A, S4 Table). These analyses showed changes in gene expression that were comparable to the RNAseq findings, though the fold-changes tended to higher by RNAseq (S4 Table). Comparable differences between RNAseq and RT-qPCR were also observed for the previously evaluated genes with established roles in spermatogonial cell biology (S4 Table).

**Fig 7:**
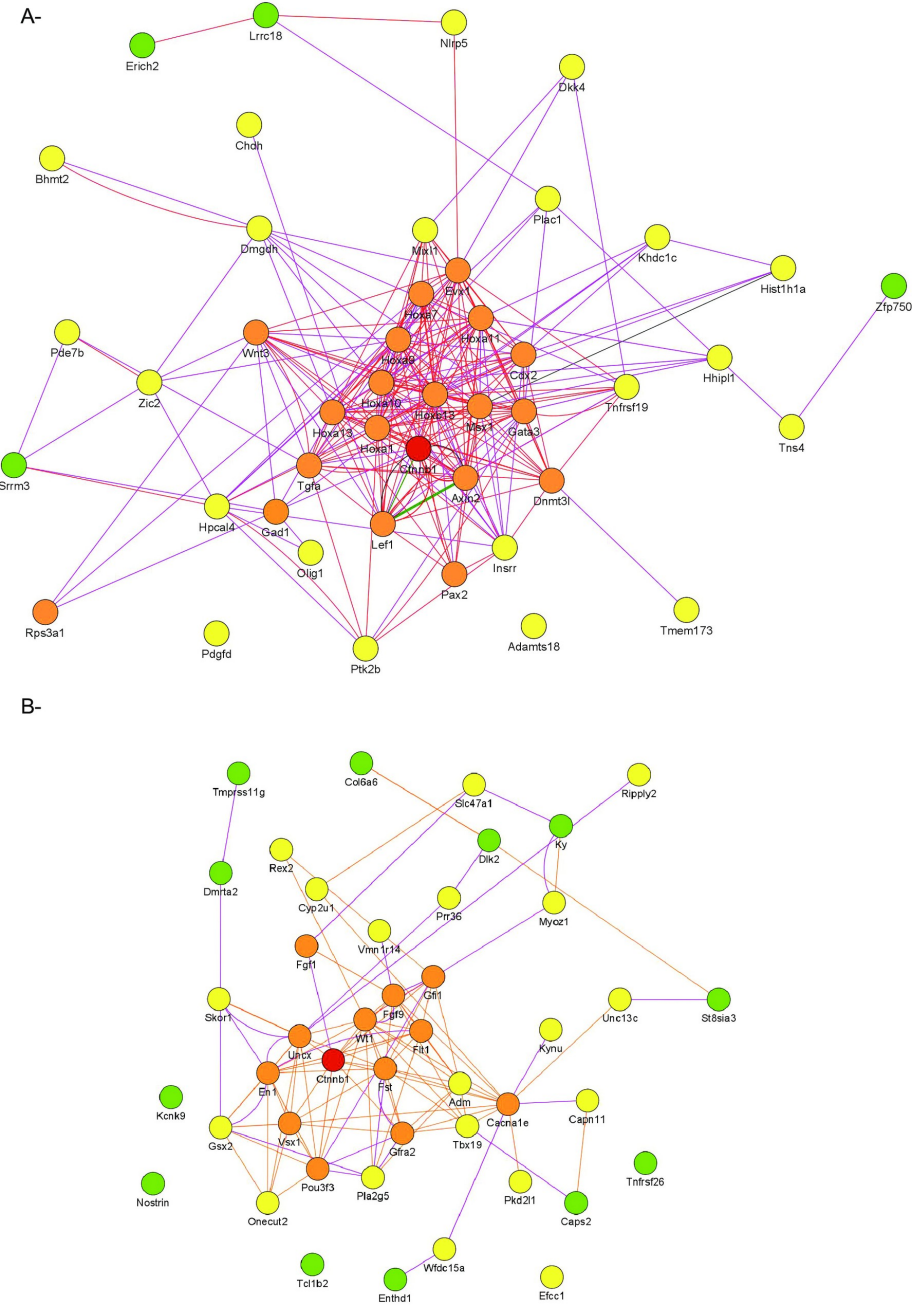
CTNNB1 regulates an important network of genes in spermatogonia. **A)** Gene network analysis of the 50 most upregulated genes in Thy1-positive cell clusters derived from *Rosa*-Δ*Ctnnb1* mice. **B)** Gene network analysis of the 50 most downregulated genes in Thy1-positive cell clusters derived from *Rosa*-Δ*Ctnnb1* mice. Lines, orange = predicted interactions, purple = co-expression, green = genetic interaction, red = pathway, black = physical interaction. Circle; orange = first neighbor, yellow = second neighbor, green = third neighbor.

To study the biological processes affected by CTNNB1 overexpression in *Rosa*-Δ*Ctnnb1* cluster cells, both upregulated and downregulated genes were subjected to gene ontology analysis using the Metascape gene annotation and analysis resources. This showed that the main biological processes in which the upregulated and downregulated genes are involved are cell proliferation and tissue development (Fig 6B and 6C). Finally, to obtain a better understanding of the relationships between genes affected by CTNNB1 stabilization, a functional protein association network was generated for the 50 most highly upregulated and downregulated genes identified by RNAseq (Fig 7A and 7B). The majority of the identified genes had previously-identified or predicted interactions with CTNNB1, suggesting that, in spermatogonial cluster cells, CTNNB1 exerts its actions via interaction networks similar to those occurring in other cell types.

## Discussion

Over the last 8 years, several *in vitro* and *in vivo* models have been used to determine the roles of the WNT/CTNNB1 signaling pathway in SSCs and undifferentiated spermatogonia, with some models associating WNT/CTNNB1 with the differentiation of SSCs [13, 14, 21] and others suggesting that WNT/CTNNB1 is important for the proliferation of undifferentiated spermatogonia [18–20]. In this study, we revisited the Δ*Ctnnb1* mouse model to specifically evaluate SSC activity in the context of sustained CTNNB1 signaling.

The Δ*Ctnnb1* mouse model was previously studied by two groups [19, 20]. Similar to the current study, Kumar et al. [19] reported that the loss of spermatogenesis and testicular atrophy became detectable after three to four months in Δ*Ctnnb1* mice. This contrasts with the findings of Chassot et al. [20], who noted such changes as early as 15 dpp. The reasons underlying this discrepancy are unclear, as the mice in all three studies were on a mixed 129/Sv X C57BL/6 genetic background. Kumar et al. further showed that the loss of spermatogenesis was associated with a decrease in spermatogonial cell proliferation, followed by a block in meiotic progression [19]. They also concluded that the SSC population was not affected, as spermatogenesis was able to recover after chemical ablation [19]. This contrasts with our conclusion that loss of SSC activity is also partly responsible for the loss of spermatogenesis in the Δ*Ctnnb1* model. This difference may be because we evaluated SSC activity in 8-month-old animals, compared to Kumar et al. who depleted germ cells in animals of five weeks of age and evaluated spermatogenesis recovery in 19-week-old animals [19]. This age might have been too early to observe clear SSC depletion. We also used germ cell transplantation (considered the gold standard to evaluate SSC activity [24]) and Thy1+ cell cluster formation to assess SSC populations, whereas Kumar et al. [19] relied on the analysis of biological markers that are usually also expressed in undifferentiated spermatogonia [41]. Differences in methods may therefore have permitted us to observe more subtle effects on SSC activity in the present study.

A second mouse model featuring constitutive activation of CTNNB1 in male germ cells (*Ctnnb1*^tm1Mmt/tm1Mmt^; *Nanos3*^cre/+^) has also been reported [21]. In this model, a spermatogenesis defect occurred between 2 and 4 months of age, which was associated with a reduction of GFRA1+ cells and proposed to be caused by the differentiation of the SSCs [21]. These results are in line with our findings that the expression of markers of SSC maintenance was downregulated in *Rosa*-Δ*Ctnnb1* clusters and that expression of markers of undifferentiated progenitor spermatogonia was upregulated, again suggesting that CTNNB1 signaling skews SSC fate toward their transition to undifferentiated progenitor spermatogonia. Our findings are also broadly compatible with a previous study that used mouse models for lineage tracing (*Axin2*^CreERT2/+^;Rosa26^rbw/+^) and inactivation of *Ctnnb1* (*Axin2*^CreERT2/+^;*Ctnnb1*^tm2Kem/-^) [18]. The latter report showed that *Ctnnb1* is important for the proliferation of undifferentiated spermatogonia already committed to differentiation, but does not play a role in the maintenance (i.e., self-renewal) of the SSC population [18]. It is important to note that we did not evaluate later stages of spermatogenesis in our study, as Kumar et al. did an extensive evaluation of meiotic progression [19]. We believe that their study, in combination with our own, indicate that CTNNB1 signaling is important for numerous steps of spermatogenesis progression.

Two previous studies evaluated the effects of the activation of CTNNB1 on SSCs using culture models similar to the ones used in the present report. In the first, LiCl treatment (which activates CTNNB1) significantly decreased SSC numbers present in germ cell clusters [13], which is in agreement with our *in vivo* and *ex vivo* findings in the Δ*Ctnnb1* model. However, a second study employed WNT3a to activate CTNNB1 in germ cell clusters, resulting in an increase in SSC numbers. This seemingly contradictory finding was subsequently explained when it was shown that, although the subset of cells expressing CTNNB1 had reduced SSC activity (as expected), WNT3a had acted indirectly on SSCs by stimulating the proliferation of cells committed to differentiation, thereby creating an *in vitro* environment supportive of SSC activity [14]. In addition, SSCs have been shown to express shisa family member 6 (SHISA6) [21], a WNT inhibitor that may act to retain the frizzled family of WNT receptors in the endoplasmic reticulum [42]. SHISA6 may therefore render SSCs refractory to WNTs, but cannot protect SSCs from the deleterious effects of CTNNB1 signaling when activated by a WNT-independent mechanism, such as by LiCl treatment or by genetic means in the Δ*Ctnnb1* model.

Although several studies have evaluated the effect of CTNNB1 on spermatogenesis, a global analysis of the genes regulated by CTNNB1 in undifferentiated spermatogonia has never been reported; the only previous high throughput analysis having been performed on whole testis [19]. The RNAseq experiment detailed in this study revealed that the expression of more than 900 genes were either upregulated or downregulated by at least two-fold by the stabilization of CTNNB1 in undifferentiated spermatogonial cell clusters, suggesting that CTNNB1 is an important regulator of biological processes in spermatogonial cells. Among the ten most highly overexpressed genes identified in the Δ*Ctnnb1* cluster cells are four members of the *Hoxa* gene cluster (*Hoxa9*, *10*, *11*, *13*), with *Hoxa7* and *Hoxa1* also included among the 25 most upregulated genes. Numerous Hox family members have been associated with mammalian sexual development (reviewed in [43]), including *Hoxa11* which have been associated with cryptorchidism [44]. However, the potential role of the *Hoxa* gene cluster in early stages of spermatogenesis has never been evaluated. It was however shown that regulation of the expression of *Hoxa* cluster members is important for determining the cell fate of osteoblasts [45] and regulating definitive hematopoiesis [46], perhaps indicating that members of the *Hoxa* cluster could also act synergistically to regulate the fate of undifferentiated spermatogonia.

Also among genes upregulated in Δ*Ctnnb1* cluster cells are glutamate decarboxylase 1 (*Gad1*), Msh homeobox 1 (*Msx1*), DNA (cytosine-5)-methyltransferase 3-like (*Dnmt3l*) and Paired box 2 (*Pax2*). *Gad1* and *Msx1* are both known downstream targets of CTNNB1 [47, 48], and are respectively involved in the inhibition of SSC proliferation [49] and the promotion of meiosis initiation [50, 51]. Roles for *Dnmt3l* have been suggested both in the maintenance of SSCs [52] and in progression through meiosis [53, 54]. Though the role of *Pax2* (the second most highly upregulated gene in our data) in spermatogenesis has not been properly evaluated, it has been shown that PAX2 binding sites are present in the promoters of an important network of genes whose expression is increased during the transition of spermatogonia to spermatocytes [55]. The increase in the expression of these genes suggests that CTNNB1 signaling might not only be important for the transition of SSCs to undifferentiated progenitor spermatogonia, but may also play a role in the initiation of meiosis.

Among the genes shown to be downregulated in Δ*Ctnnb1* cluster cells, *Eomes* and *Vsx1* are known targets of CTNNB1 [56–58] and are expressed in SSCs [6, 39]. Wilms tumors 1 (WT1) and CTNNB1 are known to antagonize each other’s activity in several contexts [59], including in the developing gonads [25, 60]. Although WT1 is better known for its roles in Sertoli cells, it was previously shown that it is also expressed in embryonic male germ cells and is important for their proliferation or survival [61], suggesting that WT1 could also have a role in SSCs postnatally. The role of engrailed 1 (*En1*, the most highly downregulated gene identified in our RNAseq analysis) in spermatogenesis has not yet been evaluated. However, a recent study showed that the EN1 motif is present at a high frequency in accessible regions of chromatin in human pachytene spermatocytes, but not in differentiating spermatogonia [62]. *En1* regulation by CTNNB1 could therefore help reconcile the results of this study with the results obtained by Kumar et al. [19], as stabilization of CTNNB1 would first antagonize SSC activity and promote entry of spermatogonia into meiosis, but would then block progression through later stages of meiosis by inhibiting *En1*.

In summary, although several reports have provided evidence for the role of CTNNB1 in SSCs and undifferentiated spermatogonia, none have evaluated the global effects of CTNNB1 in these cell populations. In this study we employed gene targeting, germ cell transplant, primary cell culture and RNAseq technologies to more directly look into the role of CTNNB1 signaling in controlling SSC activity. Our data identify several candidate genes which may act downstream of CTNNB1 in spermatogonia, and support findings from previous studies that CTNNB1 signaling promotes the transition of SSCs to undifferentiated progenitor spermatogonia at the expense of their maintenance. Our study also demonstrates the value of revisiting published models using different techniques to better understand the mechanisms of action of genes of interest.

## Supporting information

S1 Table List of the top 50 upregulated genes in undifferentiated spermatogonial aggregates (clusters) derived from *Rosa*-Δ*Ctnnb1* mice. (DOCX)

S2 Table List of the top 50 downregulated genes in undifferentiated spermatogonial aggregates (clusters) derived from *Rosa*-Δ*Ctnnb1* mice. (DOCX)

S3 Table (XLSX)

S4 Table Comparison of fold-change in mRNA levels (*Rosa-ΔCtnnb1* vs *Rosa-control*) for selected genes, as determined by RNAseq and RT-qPCR. (DOCX)

S1 Fig (PDF)
