# Supplementary material for: Constitutive activation of CTNNB1 results in a loss of spermatogonial stem cell activity in mice
Source: PLoS One. 2021 May 20;16(5):e0251911. doi: 10.1371/journal.pone.0251911 (PMC8136708; doi:10.1371/journal.pone.0251911)
Supplement: S1 Table — (DOCX) [file pone.0251911.s001.docx]

**S1 Table. List of the top 50 upregulated genes in undifferentiated spermatogonial aggregates (clusters) derived from *Rosa*-Δ*Ctnnb1* mice.**

| **Gene** | **Fold-change** | **padj** |
| --- | --- | --- |
| \| Hoxa11 \| \| --- \| \| Pax2 \| \| Hoxa13 \| \| Evx1 \| \| Hoxa10 \| \| 4930412O13Rik \| \| Dkk4 \| \| Lef1 \| \| Gad1 \| \| Hoxa9 \| \| Dmgdh \| \| Cdx2 \| \| Gata3 \| \| Bhmt2 \| \| Tns4 \| \| Mixl1 \| \| Khdc1c \| \| Lrrc18 \| \| Tgfa \| \| Pnp2 \| \| 9530053A07Rik \| \| Pdgfd \| \| Hoxa7 \| \| Hoxa1 \| \| Nlrp5 \| \| Zfp750 \| \| Olig1 \| \| Plac1 \| \| Axin2 \| \| Erich2 \| \| Ptk2b \| \| Adamts18 \| \| Hoxb13 \| \| Hist1h2bj \| \| Chdh \| \| Pde7b \| \| Otud6a \| \| Msx1 \| \| Dnmt3l \| \| Rps3a1 \| \| Tmem173 \| \| Hhipl1 \| \| Tnfrsf19 \| \| Hist1h1a \| \| Sp7 \| \| Hpcal4 \| \| Nxph3 \| \| Wnt3 \| \| Insrr  Aqp3 \| | \| 584.21 \| \| --- \| \| 508.41 \| \| 256.56 \| \| 149.13 \| \| 143.23 \| \| 68.38 \| \| 63.95 \| \| 62.98 \| \| 62.75 \| \| 61.56 \| \| 60.20 \| \| 36.39 \| \| 35.76 \| \| 29.47 \| \| 23.47 \| \| 22.16 \| \| 19.12 \| \| 18.69 \| \| 18.56 \| \| 16.19 \| \| 14.80 \| \| 14.29 \| \| 14.06 \| \| 13.69 \| \| 13.61 \| \| 12.72 \| \| 12.60 \| \| 12.57 \| \| 11.75 \| \| 11.44 \| \| 10.65 \| \| 10.00 \| \| 9.57 \| \| 9.15 \| \| 8.99 \| \| 8.89 \| \| 8.70 \| \| 8.51 \| \| 8.16 \| \| 7.92 \| \| 7.82 \| \| 7.78 \| \| 7.75 \| \| 7.53 \| \| 7.51 \| \| 7.45 \| \| 7.43 \| \| 7.34 \| \| 7.33 \|   7.29 | \| 2.98 e-13 \| \| --- \| \| 2.48 e-33 \| \| 1.79 e-13 \| \| 3.12 e-08 \| \| 1.63 e-25 \| \| 4.13 e-05 \| \| 6.02 e-05 \| \| 0 \| \| 8.04 e-139 \| \| 1.77 e-13 \| \| 2.33 e-05 \| \| 4.51 e-12 \| \| 2.47 e-78 \| \| 0.00011 \| \| 1.27 e-05 \| \| 1.73 e-82 \| \| 1.75 e-07 \| \| 1.55 e-56 \| \| 2.10 e-12 \| \| 5.91 e-12 \| \| 0.00061 \| \| 8.25 e-05 \| \| 2.13 e-28 \| \| 1.90 e-07 \| \| 6.56 e-13 \| \| 2.39 e-28 \| \| 5.70 e-06 \| \| 0.00027 \| \| 7.91 e-268 \| \| 7.06 e-40 \| \| 1.64 e-36 \| \| 3.99 e-11 \| \| 2.72 e-25 \| \| 5.89 e-09 \| \| 4.09 e-07 \| \| 1.52 e-15 \| \| 7.50 e-22 \| \| 1.33 e-54 \| \| 8.51 e-13 \| \| 0 \| \| 5.12 e-62 \| \| 2.22 e-18 \| \| 1.30 e-92 \| \| 4.87 e-27 \| \| 1.24 e-07 \| \| 4.95 e-07 \| \| 1.61 e-11 \| \| 3.97 e-41 \| \| 2.66 e-06  0.000762 \| |
