# Supplementary material for: Constitutive activation of CTNNB1 results in a loss of spermatogonial stem cell activity in mice
Source: PLoS One. 2021 May 20;16(5):e0251911. doi: 10.1371/journal.pone.0251911 (PMC8136708; doi:10.1371/journal.pone.0251911)
Supplement: S2 Table — (DOCX) [file pone.0251911.s002.docx]

**S2 Table. List of the top 50 downregulated genes in undifferentiated spermatogonial aggregates (clusters) derived from *Rosa*-Δ*Ctnnb1* mice.**

| **Gene** | **Fold-change** | **padj** |
| --- | --- | --- |
| \| En1 \| \| --- \| \| Vmn1r14 \| \| Kynu \| \| Col6a6 \| \| Ky \| \| Wt1 \| \| Ripply2 \| \| Uncx \| \| Onecut2 \| \| Dmrta2 \| \| Capn11 \| \| Skor1 \| \| Gsx2 \| \| Kcnk9 \| \| Vsx1 \| \| Rex2 \| \| Cyp2u1 \| \| Tnfrsf26 \| \| Fgf1 \| \| Cdc42ep3 \| \| St8sia3 \| \| Flt1 \| \| Adh6b \| \| Unc13c \| \| Pou3f3 \| \| Tbx19 \| \| Enthd1 \| \| Dlk2 \| \| Fgf9 \| \| Zfp978 \| \| Myoz1 \| \| Prr36 \| \| Ubap1l \| \| Pla2g5 \| \| Gfi1 \| \| Tcl1b2 \| \| Adm \| \| Caps2 \| \| Slc47a1 \| \| Tmprss11g \| \| Fst \| \| Cacna1e \| \| Efcc1 \| \| Slc38a3 \| \| Wfdc15a \| \| Pkd2l1 \| \| Aox4 \| \| 1700012B07Rik \| \| Unc80 \|   Izumo1r | \| -1335.51 \| \| --- \| \| -199.63 \| \| -71.54 \| \| -71.28 \| \| -68.83 \| \| -63.08 \| \| -42.16 \| \| -28.87 \| \| -26.60 \| \| -17.50 \| \| -16.50 \| \| -13.03 \| \| -9.78 \| \| -9.49 \| \| -8.96 \| \| -8.85 \| \| -7.79 \| \| -7.10 \| \| -6.82 \| \| -6.80 \| \| -6.76 \| \| -6.68 \| \| -6.62 \| \| -6.12 \| \| -5.67 \| \| -5.52 \| \| -5.40 \| \| -5.39 \| \| -5.22 \| \| -5.19 \| \| -5.06 \| \| -4.69 \| \| -4.64 \| \| -4.59 \| \| -4.58 \| \| -4.53 \| \| -4.53 \| \| -4.52 \| \| -4.42 \| \| -4.40 \| \| -4.39 \| \| -4.36 \| \| -4.35 \| \| -4.29 \| \| -4.28 \| \| -4.27 \| \| -4.14 \| \| -4.08 \| \| -3.97 \| \| -3.92 \| | \| 1.36 e-44 \| \| --- \| \| 6.80 e-09 \| \| 2.60 e-05 \| \| 1.27 e-28 \| \| 8.99 e-93 \| \| 4.04 e-33 \| \| 0.00020 \| \| 4.69 e-14 \| \| 7.26 e-296 \| \| 3.02 e-36 \| \| 1.73 e-05 \| \| 2.01 e-25 \| \| 1.47 e-06 \| \| 7.78 e-56 \| \| 1.02 8e-35 \| \| 1.28 e-05 \| \| 0 \| \| 1.86 e-29 \| \| 3.30 e-05 \| \| 1.34 e-157 \| \| 9.11 e-46 \| \| 4.15 e-246 \| \| 0.00205 \| \| 3.72 e-12 \| \| 8.86 e-69 \| \| 3.41 e-05 \| \| 1.56 e-05 \| \| 1.02 e-07 \| \| 1.80 e-71 \| \| 1.13 e-09 \| \| 0.00015 \| \| 0.00010 \| \| 2.49 e-05 \| \| 9.60 e-13 \| \| 0.00035 \| \| 0.01754 \| \| 2.06 e-31 \| \| 5.42 e-16 \| \| 1.57 e-118 \| \| 0.02274 \| \| 1.24 e-280 \| \| 3.44 e-32 \| \| 1.76 e-06 \| \| 1.71 e-30 \| \| 7.96 e-10 \| \| 2.00 e-05 \| \| 1.87 e-14 \| \| 2.70 e-05 \| \| 0.01243 \| \| 6.44 e-05 \| |
