# Supplementary material for: Constitutive activation of CTNNB1 results in a loss of spermatogonial stem cell activity in mice
Source: PLoS One. 2021 May 20;16(5):e0251911. doi: 10.1371/journal.pone.0251911 (PMC8136708; doi:10.1371/journal.pone.0251911)
Supplement: S4 Table — (DOCX) [file pone.0251911.s004.docx]

**S4 Table. Comparison of fold-change in mRNA levels (*Rosa-ΔCtnnb1* vs *Rosa-control*) for selected genes, as determined by RNAseq and RT-qPCR.**

| **Gene** | **RT-qPCR fold-change** | **RNAseq fold-change** |
| --- | --- | --- |
| *Axin2*  *Bcl6b*  *En1*  *Eomes1*  *Gfra1*  *Hoxa9*  *Kit*  *Lef1*  *Msx1*  *Onecut2*  *Pax2*  *Plzf*  *Rarg*  *Sohlh1*  *Vsx1*  *Wt1* | 13.10  -2.13  -24.42  -2.90  -1.75  58.12  2.54  40.57  5.33  -1.84  85.91  -1.90  1.36  1.39  -2.52  -40.79 | 11.75  -2.23  -1335.51  -3.22  -1.61  61.56  2.26  62.98  8.51  -26.60  508.41  -1.81  1.88  1.82  -8.96  -63.08 |
