# Supplementary material for: Constitutive activation of CTNNB1 results in a loss of spermatogonial stem cell activity in mice
Source: PLoS One. 2021 May 20;16(5):e0251911. doi: 10.1371/journal.pone.0251911 (PMC8136708; doi:10.1371/journal.pone.0251911)

Fig S1, raw_image

PCR products were analyzed using a 2% agarose gel containing ethidium bromide and photographed under UV illumination.

Figure 1A was generated from this gel

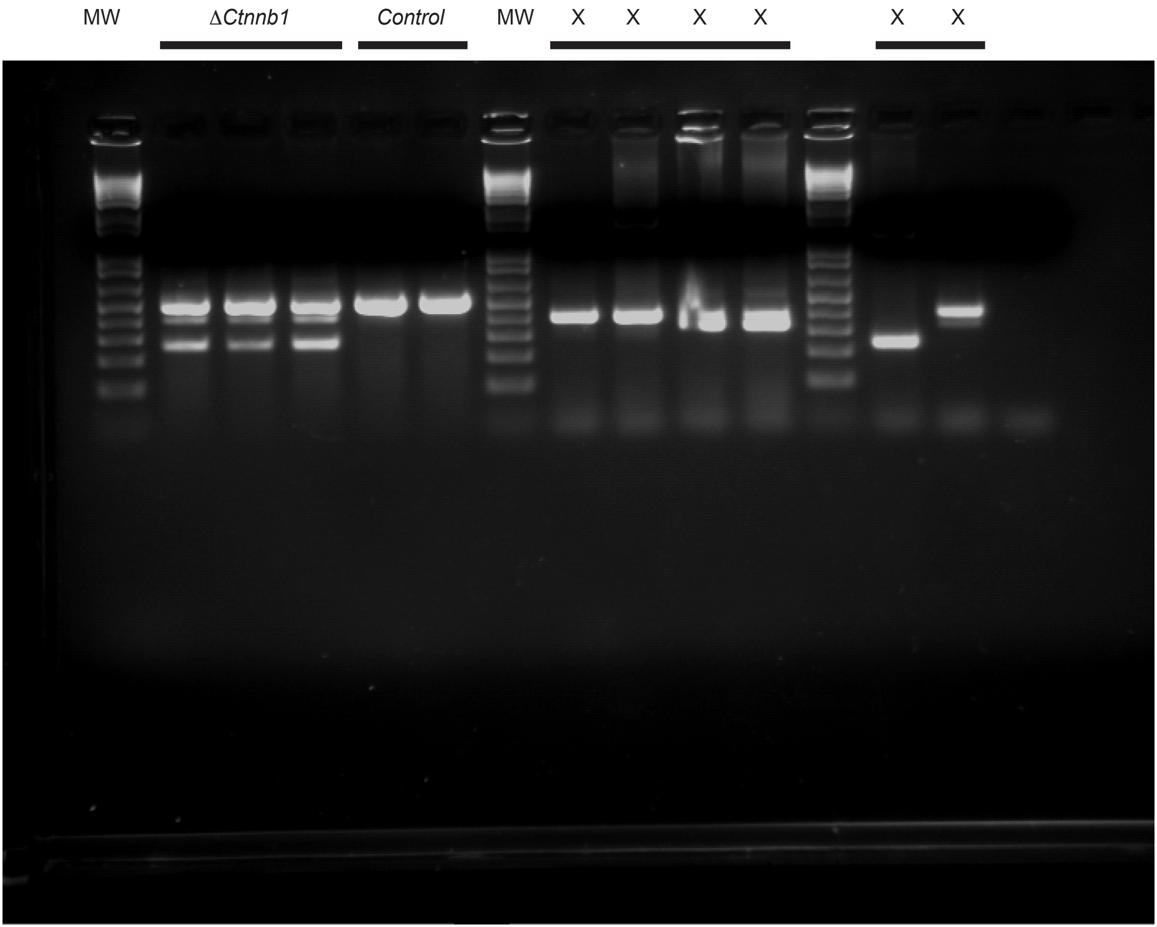

Supplement: S1 Fig — (PDF) [file pone.0251911.s005.pdf]
